# Evaluating the Predictive Capability of Radiomics Features of Perirenal Fat in Enhanced CT Images for Staging and Grading of UTUC Tumours Using Machine Learning

**DOI:** 10.3390/cancers17071220

**Published:** 2025-04-04

**Authors:** Abdulrahman Al Mopti, Abdulsalam Alqahtani, Ali H. D. Alshehri, Chunhui Li, Ghulam Nabi

**Affiliations:** 1 Centre for Medical Engineering and Technology, School of Medicine, University of Dundee, Dundee DD1 9SY, UK; axalqahtani@dundee.ac.uk (A.A.); g.nabi@dundee.ac.uk (G.N.); 2 Radiology Department, College of Applied Medical Sciences, Najran University, Najran 55461, Saudi Arabia; ahzafer@nu.edu.sa; 3 School of Science and Engineering, University of Dundee, Dundee DD1 4HN, UK; c.li@dundee.ac.uk

**Keywords:** CT urogram, machine learning, radiomics, texture analysis, perirenal fat

## Abstract

**Background:** Upper tract urothelial carcinoma (UTUC) often presents with aggressive behaviour, demanding accurate preoperative assessment to guide management. Radiomics-based approaches have shown promise in extracting quantitative features from imaging, yet few studies have explored whether perirenal fat (PRF) radiomics can augment tumour-only models. **Methods:** A retrospective cohort of 103 UTUC patients undergoing radical nephroureterectomy was analysed. Tumour regions of interest (ROI) and concentric PRF expansions (10–30 mm) were segmented from computed tomography (CT) scans. Radiomic features were extracted using PyRadiomics, filtered by correlation and intraclass correlation coefficients, and integrated with clinical variables (e.g., age, BMI, multifocality). Multiple machine learning models, including MLPClassifier and CatBoost, were evaluated via repeated cross-validation. Performance was assessed using the area under the ROC curve (AUC), sensitivity, specificity, F1-score, and DeLong tests. **Results:** The best tumour grade model (AUC = 0.961) merged tumour-derived features with a 10 mm PRF margin, exceeding PRF-only (AUC = 0.900) and tumour-only (AUC = 0.934) approaches. However, the improvement over tumour-only was not always statistically significant. For stage prediction, combining tumour and 15 mm PRF features yielded the top AUC of 0.852, surpassing the tumour-alone model (AUC = 0.802) and outperforming PRF-only (AUC ≤ 0.778). PRF features provided an additional predictive value for both grade and stage models. **Conclusions:** Integrating PRF radiomics with tumour-based analyses enhances predictive accuracy for UTUC grade and stage, suggesting that the tumour microenvironment contains complementary imaging cues. These findings, pending external validation, support the potential for radiomics-driven risk stratification and personalised treatment planning in UTUC.

## Simple Summary

This retrospective study investigates the diagnostic and prognostic utility of perirenal fat (PRF) radiomic features in upper tract urothelial carcinoma (UTUC). Building on prior work that demonstrated promising outcomes using tumour-only radiomics, the current research evaluates whether integrating PRF features with tumour-based models further improves predictions of tumour grade and stage, potentially enhancing risk stratification and clinical decision-making.

## 1. Introduction

Upper tract urothelial carcinoma (UTUC) is a rare but aggressive malignancy that accounts for approximately 5–10% of all urothelial cancers [1]. Despite its low incidence, UTUC has a high potential for recurrence and progression, with a 5-year survival rate of less than 50% for advanced-stage disease [2,3]. The clinical management of UTUC remains challenging due to the difficulties in early diagnosis, accurate staging, and predicting treatment response [4,5].

Current diagnostic methods for UTUC, such as urine cytology and ureteroscopic biopsy, have limitations in terms of sensitivity and specificity [6,7]. Imaging techniques, particularly computed tomography (CT) and magnetic resonance imaging (MRI), play a crucial role in the evaluation of UTUC [8]. However, conventional imaging analysis relies on subjective interpretation and may not fully capture the complex tumour characteristics and microenvironment [9,10].

In addition to CT and MRI, diagnostic ureteroscopy plays an important role in characterising high-risk UTUC, as evidenced by a recent multi-institutional collaborative analysis (ROBUUST Collaborative Group) [11]. Their findings highlight how a preoperative ureteroscopic evaluation can refine both decision-making and prognosis in patients with aggressive disease, potentially leading to earlier radical interventions. However, a ureteroscopic biopsy is invasive and may still provide limited tissue sampling or understage certain lesions. This underscores the need for complementary, non-invasive imaging biomarkers that can more fully capture tumour aggressiveness and the surrounding microenvironment.

In recent years, there has been growing interest in the role of the tumour microenvironment (TME) in cancer progression and treatment response [12]. The TME, which includes the surrounding blood vessels, immune cells, fibroblasts, and adipose tissue, has been shown to influence tumour growth, invasion, and metastasis [13,14]. In particular, the PRF has emerged as a critical component, particularly in diagnosing and prognosticating UTUC [15]. Studies indicate that the characteristics of PRF, such as thickness and infiltration by the tumour, can provide valuable prognostic and diagnostic information in upper urinary tract cancer [15,16,17,18,19].

A previous study by our group (Alqahtani et al., 2024) demonstrated the potential of radiomics-based machine learning approaches in predicting tumour histological grade and Tumour, Node, Metastases (TNM) stage in UTUC using features extracted from the tumour region of interest (ROI). The study achieved high accuracy in predicting tumour grade (AUC 0.94) and moderate accuracy for tumour stage (AUC 0.75) [20]. Building upon these findings, the current study aims to explore the diagnostic and prognostic value of the PRF surrounding the tumour. This approach could potentially provide complementary information to direct tumour analysis, offering a more comprehensive assessment of UTUC.

Radiomics, an emerging field that involves the extraction of quantitative features from medical images, has shown promise in capturing the heterogeneity and complexity of the TME [9,21,22]. By applying advanced image analysis techniques and machine learning algorithms, radiomics has the potential to provide non-invasive biomarkers for diagnosis, prognosis, and treatment response prediction [9].

Given UTUC’s anatomical location and the potential influence of PRF on tumour behaviour, this study hypothesises that a radiomics analysis of the PRF could provide valuable diagnostic and prognostic information. This study aims to investigate the relationship between PRF radiomics features and key clinical outcomes in UTUC, including tumour stage and grade. By leveraging advanced imaging analytics and machine learning techniques, we seek to develop a novel, non-invasive approach to improve risk stratification and personalised treatment planning for patients with UTUC.

## 2. Materials and Methods

### 2.1. Adherence to Guidelines and Ethical Considerations

This study adheres to the CheckList for EvaluAtion of Radiomics Research (CLEAR) guidelines and has received ethical approval from NHS Tayside (ref IGTCAL12931) (Supplementary File S1) [23]. A waiver for consent was granted due to the retrospective nature of the study, ensuring that all the data handling processes adhered to the approved guidelines for patient data confidentiality and integrity.

### 2.2. Patient Cohort Characteristics

Data was collected by searching the database of our institution from January 2000 to December 2022. The study comprised individuals who underwent a radical nephroureterectomy during this period. From the institutional database (n = 256), patients were selected based on inclusion criteria: available CT urogram datasets, adherence to imaging protocols, histologically confirmed UTUC, and no prior endoscopic UTUC treatment before CT. Exclusions (n = 150) were due to non-contrast CT scans (n = 114), poor image quality (n = 12), pre-CT treatments (n = 5), and incomplete data (n = 19). Additional cases were excluded after segmentation due to inadequate inter-reader agreement, resulting in 103 patients for final analysis. Nephroureterectomy specimens served as the histopathological reference standard.

Demographics showed a median age of 74 years (range: 49–93), with 59% males and 41% females. Most patients (78%) were current or former smokers. Tumours were classified as low-grade (I–II, n = 30) or high-grade (III–IV, n = 73), and staged as early (Ta–T1, n = 58) or advanced (T2–T4, n =4 5). Tumours were located in the renal pelvis (48%) or ureter (52%), with a mean size of 1.97 ± 0.83 cm. Table 1 presents comprehensive patient characteristics.

### 2.3. CT Imaging Protocol

All CT scans were conducted following a standardised protocol on a 64-slice multidetector CT scanner (Somatom Definition AS, Siemens Healthineers, Erlangen, Germany). The protocol included acquisitions in the non-contrast phase, the nephrographic phase (approximately 100 s post-contrast), and the excretory phase (around 10 min post-contrast). An intravenous injection of 100 mL Omnipaque 300 (GE Healthcare, Chicago, IL, USA) was administered at a rate of 3 mL/s. Images were reconstructed with a 1 mm slice thickness and a 0.7 mm overlap.

### 2.4. Image Segmentation

Two independent readers performed tumour segmentation using the ’grow-from-seeds’ tool in 3D Slicer (version 5.2.2). A Dice Similarity Coefficient (DSC) threshold of 0.8 was applied to ensure at least 80% overlap between their segmentations. Of the original cases, 88 immediately satisfied the DSC threshold of ≥0.8. Among the remaining 18 cases, readers resolved disagreements in 15 instances. However, three cases did not reach a consensus and were excluded.

A semi-automated method was developed for PRF segmentation, expanding the tumour segmentation by 5, 10, 15, 20, 25, and 30 mm around the tumour and both kidneys to encompass peritumoral fat. A total of six ROIs were generated. A threshold range of −30 to −200 Hounsfield units (HU) was applied to segment only fat and exclude adjacent organs. Morphological operations were performed on each segmentation to remove small dots and fill small holes.

### 2.5. Feature Extraction and Analysis

Radiomic features were extracted using PyRadiomics (v3.0.1) in Python, broadly adhering to IBSI recommendations for feature definitions, with some software-specific deviations as documented in the PyRadiomics manual.

All CT images were resampled to isotropic 1 × 1 × 1 mm spacing via B-spline interpolation and then normalized (scale = 500). A fixed bin width of 25 HU was used for intensity discretisation, and outliers were removed at 1.5σ. To capture multi-scale textural patterns across different feature spaces, multiple filters were applied including wavelet, Laplacian of Gaussian, gradient, and local binary pattern. First-order statistics, texture, and shape features were extracted from both original and filtered images. This comprehensive filter application yielded a total of 1409 features per ROI.

The Synthetic Minority Over-sampling Technique (SMOTE) was employed for the low-grade class to mitigate class imbalance.

### 2.6. Feature Stability and Selection

Feature selection employed a multi-step process: normalisation of numerical variables, removal of highly correlated features (Pearson correlation > 0.9), and exclusion of features with ICC < 0.75. Recursive feature elimination with cross-validation (RFECV) guided by SHAP values was then applied to identify the optimal feature subset for each ROI.

### 2.7. Machine Learning Models and Implementation

Predictive models were developed using various machine learning algorithms:

- Linear Models: Logistic Regression (LR), Support Vector Classifier (SVC), Quadratic Discriminant Analysis (QDA).
- Ensemble Methods: Random Forest Classifier (RFC), Extra Trees Classifier (ETC), Gradient Boosting Classifier (GBC), LightGBM Classifier (LGBM), CatBoost Classifier, and AdaBoost Classifier.
- Neural Networks: Multilayer Perceptron (MLPClassifier).
- Instance-Based Methods: K-Nearest Neighbours (KNN).
- Tree-Based Methods: Decision Tree Classifier (DTC).

Hyperparameters were optimised using grid search with 5-fold stratified cross-validation. Implementation utilised scikit-learn (version 0.24.2) for most classifiers, with dedicated libraries for specialised algorithms. Performance evaluation employed 5-fold repeated cross-validation with metrics including AUC, sensitivity, specificity, and F1-score.

### 2.8. Statistical Analysis

Statistical analyses were conducted, including the calculation of 95% confidence intervals (CI) using bootstrapping with 1000 iterations. DeLong’s test assessed statistical significance between AUCs from different model configurations (*p* < 0.05 considered significant). SHAP values were calculated to interpret feature importance and understand model predictions. All analyses were performed using Python 3.7.

## 3. Results

### 3.1. Segmentation Results

Our segmentation approach produced one ROI for the tumour and five separate ROIs for perirenal fat at increasing distances (10, 15, 20, 25, and 30 mm margins from the tumour boundary). Although we initially considered a 5 mm margin, inconsistent tumour-to-fat boundaries rendered that approach impractical. The semi-automatic segmentation method accurately delineated the PRF fat while effectively removing surrounding tissues.

### 3.2. Radiomic Feature Selection

From each ROI, we initially extracted 1409 radiomic features spanning intensity, shape, and texture. For the PRF ROIs, Pearson correlation eliminated 376 to 895 features per ROI due to redundancy. For the tumour ROIs, applying the ICC threshold reduced the feature set to 163 robust features. The final RFECV with SHAP analysis retained between 3 and 12 optimal features per ROI, maximising predictive performance.

### 3.3. Model Performance for Tumour Grade Prediction

**Figure 1:**
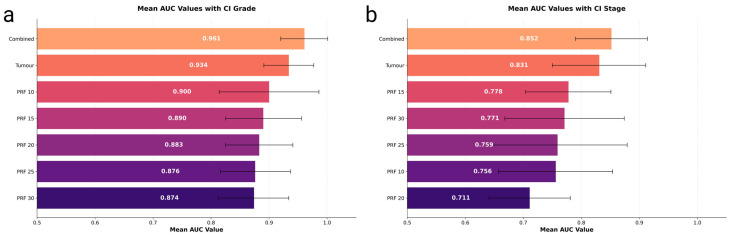
(**a**) Mean AUC (Grade)—Bar plot of mean ROC AUC (with 95% CI) for predicting tumour grade across different configurations (Tumour, PRF at 10–30 mm, and combined Tumour + PRF). (**b**) Mean AUC (Stage)—Bar plot of mean ROC AUC (with 95% CI) for predicting tumour stage (early vs. advanced) using the same configurations.

We compared seven configurations for grade prediction, each incorporating clinical variables with different feature combinations: tumour, PRF (at various margins), and combined tumour + PRF approaches. The mean AUC values for all configurations are illustrated in Figure 1a.

**Figure 2:**
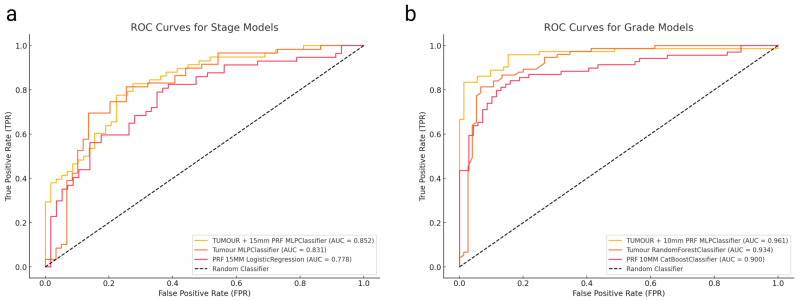
(**a**) ROC curves for histological grade prediction models, showing Tumour + 10 mm PRF, Tumour, and PRF features. (**b**) ROC curves for TNM staging prediction models, comparing Tumour + 15 mm PRF, Tumour, and PRF models.

The highest performance came from a combined model (tumour + 10 mm PRF) using MLPClassifier, achieving an AUC of 0.961 (95% CI: 0.920–1.000) with a balanced sensitivity and specificity both at 0.889. The tumour-only model reached an AUC of 0.934 (95% CI: 0.891–0.977) with a sensitivity and specificity of 0.867, while the best PRF-only approach (CatBoost at 10 mm margin) yielded 0.900 (95% CI: 0.814–0.986) with a sensitivity of 0.783 and specificity of 0.884 (Table 2). ROC curves for these three best-performing grade prediction models are plotted in Figure 2a.

When directly comparing models using the DeLong test (Table 3), integrating PRF with tumour features significantly enhanced discriminative power over PRF-alone (*p* = 0.0157). However, the test showed no statistically significant difference (*p* = 0.2252) between the tumour-only and combined tumour + PRF models, despite the numerical improvement.

**Figure 3:**
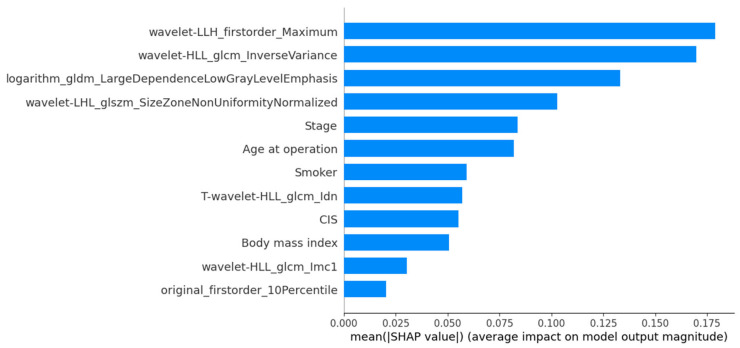
SHAP mean importance plot highlighting the most influential features in the MLPClassifier for histological grading.

**Figure 4:**
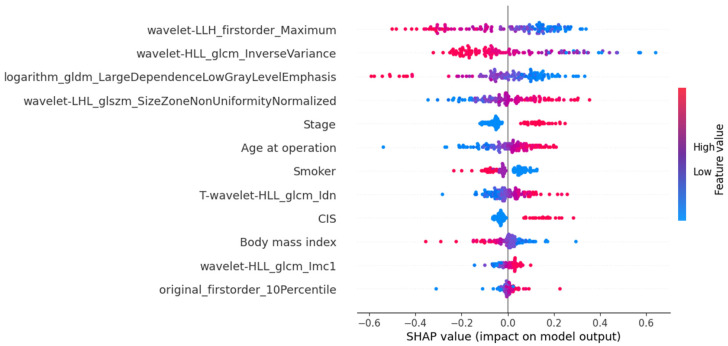
SHAP values are plotted to highlight the feature’s impact on model output in the MLPClassifier for histological grading.

The SHAP-based feature importance analysis for the best-performing grade model is shown in Figure 3, highlighting the relative contributions of different features. Figure 4 further illustrates how these features impact model output through their SHAP values.

### 3.4. Model Performance for Tumour Stage Prediction

For stage classification (early vs. advanced), the best results came from combining tumour and 15 mm PRF features (MLPClassifier), yielding an AUC of 0.852 (95% CI: 0.790–0.914) with sensitivity and specificity both at 0.776. The tumour model recorded 0.831 (95% CI: 0.750–0.911), with a sensitivity of 0.780 and specificity of 0.746, while PRF-only configurations achieved lower AUCs ranging from 0.711 to 0.778 (Table 2). The comparative performance of all configurations for stage prediction is shown in Figure 1b.

ROC curves comparing these stage prediction models are illustrated in Figure 2b. The combined approach (tumour + 15 mm PRF MLPClassifier, AUC = 0.852) showed a numerical improvement over the tumour-only model. However, the DeLong test (Table 3) indicated no statistically significant difference between these approaches (*p* = 0.565). When comparing the tumour model with the best PRF model (15 mm PRF, LogisticRegression, AUC = 0.778), the difference was close to statistical significance (*p* = 0.056), suggesting a trend toward better performance with the combined approach.

**Figure 5:**
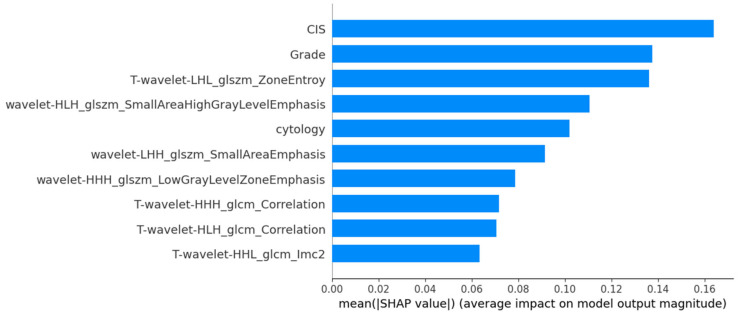
Mean SHAP importance plot highlighting the most influential features in the MLPClassifier for TNM staging.

**Figure 6:**
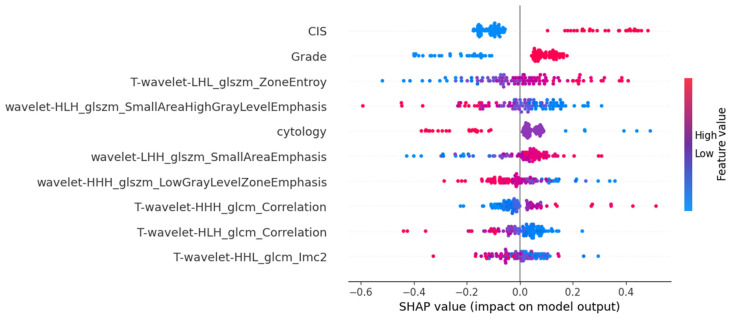
SHAP values plotted to highlight the feature’s impact on model output in the MLPClassifier for TNM staging.

The SHAP interpretations for the stage prediction models are shown in Figure 5 and Figure 6, with Figure 5 displaying the mean importance of features and Figure 6 illustrating their impact on the model’s output.

## 4. Discussion

These findings underscore the value of integrating PRF radiomic features with tumour-based radiomics in UTUC assessment. Tumour-only radiomics already provides a robust foundation, reflected in the high AUCs for grade prediction (up to 0.934). However, the combined tumour + PRF models offer incremental improvements in predicting both grade (up to 0.961) and stage (up to 0.852). Notably, PRF-only models demonstrated moderate to strong discriminatory power (AUCs up to 0.900 for grade and 0.778 for stage), highlighting that PRF harbours distinct textural cues related to tumour aggressiveness.

The DeLong tests revealed that adding PRF features significantly boosts performance over PRF-only configurations (*p* < 0.05) but does not always confer a statistically significant edge over an already strong tumour-only baseline. Nonetheless, the elevated mean AUCs (especially for grade) suggest that PRF radiomics contributes important complementary information about tumour biology and the local peritumoral environment.

Quantitative radiomic analysis highlights the prognostic value of PRF in oncology. Research such as that of Khene et al. [24] and Gill et al. [25]. showcases how CT texture analysis can predict renal tumour characteristics and differentiate renal cell carcinoma grades using metrics such as entropy. Kang Ning et al. [19] and Chung et al. [17] further demonstrated the importance of PRF thickness and stranding as independent predictors of survival in patients with metastatic renal cancer and ureteral urothelial carcinoma and proposed the inclusion of these parameters in prognostic models to improve clinical assessments and treatment strategies. These findings underscore the potential of integrating peripheral fat measurements into clinical practice to refine diagnostic and prognostic accuracy in oncology.

The research conducted by Gill et al. [25], which focused on juxtatumoural fat in renal cell carcinoma (RCC) and achieved a ROC AUC of 0.75 for predicting tumour histological grade, was extended in a study examining PRF surrounding UTUC. An automated technique for segmenting PRF, combined with clinical variables, significantly boosted the model’s performance, achieving an ROC AUC of over 0.9 for tumour grade. The precision of this automated segmentation, alongside variables such as tumour stage and BMI, provided a more comprehensive view of the disease and enhanced predictive accuracy. Moreover, the inherently aggressive and invasive nature of UTUC compared to RCC likely heightens the usefulness of PRF radiomic analysis. This distinct pathology of UTUC may account for its especially strong capacity to capture key radiomic features, as reflected in the high ROC AUC values observed in the study.

The correlation between PRF features and tumour grade can be explained by the tumour’s influence on surrounding adipose tissue. High-grade UTUC can provoke inflammatory changes in PRF, creating textural abnormalities in imaging [15,17]. Additionally, non-radiological findings—such as dense collagenous fibrosis and M2 macrophage infiltration—have been observed in high-grade UTUC, reinforcing the biological interplay between tumours and perirenal tissue. These alterations align with imaging-based fat stranding and help explain why PRF abnormalities correlate with the advanced stage as well as higher grade. A recent study demonstrated that adding PRF texture to a CT-based radiomics model improved prognostic accuracy [26]. By capturing these subtle microenvironmental cues, our combined tumour + PRF approach offers a more comprehensive assessment than tumour analysis alone.

The present study’s findings on PRF radiomics can be viewed as complementary to our previous work focusing on the tumour ROI [20]. While the tumour ROI analysis achieved a high accuracy in predicting tumour grade (AUC 0.94) and moderate accuracy for tumour stage (AUC 0.75), our current PRF radiomics approach demonstrated comparable performance for grade prediction (AUC 0. 0.961) and slightly lower accuracy for stage prediction (AUC 0.85). These findings suggest that PRF radiomics may provide additional prognostic information beyond what can be gleaned from the tumour itself. The combination of tumour ROI and PRF radiomics could potentially offer a more comprehensive assessment of UTUC, enhancing both diagnostic accuracy and prognostic capabilities. Future research should explore integrated models that leverage both tumour and PRF features to optimise UTUC characterisation and management.

Furthermore, the study by Chung et al. [17] highlights that PRF stranding (PRFS) is a secondary predictor of poor prognosis in patients with ureteral urothelial carcinoma, indicating that worse oncologic outcomes are associated with PRFS. Building on these findings, the present study applied a quantitative radiomic approach to analyse PRF textures and patterns in CT images. This strategy underscores the potential of radiomics to identify subtle imaging features such as PRF stranding, thereby enhancing the accuracy and reliability of prognostic assessments in UTUC. It not only corroborates but also extends the insights of Chung et al., supporting a more sophisticated application of PRF analysis in UTUC management.

In addition to validating and expanding on Chung et al.’s observations [17], our research introduces a new hypothesis regarding the role of PRF surrounding the tumour. Specifically, we propose that PRF has a distinct ability to predict key tumour characteristics, including tumour grade and stage. Preliminary data suggest that the radiomic signatures of PRF correlate with the biological behaviour of UTUC, aligning with prior findings that peritumoral or perirenal fat measurements can refine prognostic accuracy in renal malignancies [25,26]. By systematically integrating these features with various clinical variables, our models aim to provide oncologists with a powerful tool for personalised treatment planning, ultimately improving patient prognosis and overall disease management [3,4]. This integration of radiomic and clinical data represents a significant step forward in leveraging diagnostic imaging for refined cancer prognostication and further highlights the transformative potential of radiomics within oncology [9,21].

The clinical utility of our combined tumour + PRF model extends beyond statistical metrics to potential therapeutic decision-making. Where a ureteroscopic biopsy may be inconclusive due to sampling error [6,7], our radiomics approach could complement risk stratification by identifying patients with a high likelihood of aggressive disease, thus prompting earlier radical nephroureterectomy or consideration of neoadjuvant chemotherapy [1,2]. Similarly, preoperative identification of advanced-stage disease could guide the extent of surgery and surveillance intensity, as recommended by consensus guidelines [11]. These applications illustrate how radiomics-enhanced prognostication could meaningfully influence UTUC management strategies, bridging the gap between diagnostic imaging and personalised oncologic care [4,5]

One of the key strengths of this study is its adherence to rigorous standards and the use of advanced statistical and machine learning techniques, which ensure the accuracy and reliability of the findings. However, the single-centre design, manual segmentation of ROIs (requiring expert time), and relatively small sample size may affect the generalizability of these findings. Additionally, grade and stage imbalances could influence model training.

Future research should focus on expanding the dataset and incorporating data from multiple centres to validate and refine the predictive models. Prospective studies could provide higher-quality data and might reveal additional insights into the relationships between radiomic features and clinical outcomes. Furthermore, integrating radiomic analysis with genetic and molecular data could offer a more comprehensive understanding of tumour behaviour and response to treatment.

## 5. Conclusions

In conclusion, PRF radiomics contributes independent and complementary prognostic information in UTUC, particularly for tumour grade. The demonstrated performance gains highlight the potential for combined tumour + PRF models to improve predictive accuracy and guide clinical decision-making in UTUC management. This approach could refine risk stratification with further validation in multi-centre cohorts. Such refinement would enable more tailored therapeutic strategies and potentially improve patient outcomes.

## Figures and Tables

**Table 1:** Patient Characteristics and Tumour Features (n = 103), Stratified by Tumour Grade and Stage.

Characteristic	Overall (n = 103)	Low Grade (n = 30)	High Grade (n = 73)	Early Stage (T1) (n = 58)	Advanced Stage (T2–T4) (n = 45)
Age, median (range)	74 (49–93)	67 (49–85) *	76 (52–93) *	72 (49–88) *	77 (56–93) *
Gender, n (%)					
Male	61 (59%)	16 (53%) *	45 (62%) *	35 (60%) *	26 (58%) *
Female	42 (41%)	14 (47%) *	28 (38%) *	23 (40%) *	19 (42%) *
Smoking Status, n (%)					
Current/Former	80 (78%)	20 (67%) *	60 (82%) *	44 (76%) *	36 (80%) *
Never	23 (22%)	10 (33%) *	13 (18%) *	14 (24%) *	9 (20%) *
BMI Category, n (%)					
Normal (18.5–24.9)	34 (33%)	12 (40%) *	22 (30%) *	19 (33%) *	15 (33%) *
Overweight (25–29.9)	35 (34%)	10 (33%) *	25 (34%) *	20 (34%) *	15 (33%) *
Obese (≥30)	34 (33%)	8 (27%) *	26 (36%) *	19 (33%) *	15 (33%) *
Tumour Location, n (%)					
Renal Pelvis	49 (48%)	20 (67%) *	29 (40%) *	28 (48%) *	21 (47%) *
Ureter	54 (52%)	10 (33%) *	44 (60%) *	30 (52%) *	24 (53%) *
Carcinoma in situ, n (%)	25 (23%)	4 (13%) *	21 (29%) *	13 (22%) *	12 (27%) *
Hydronephrosis, n (%)	25 (23%)	5 (17%) *	20 (27%) *	12 (21%) *	13 (29%) *
Multifocal, n (%)	38 (35%)	8 (27%) *	30 (41%) *	18 (31%) *	20 (44%) *
Tumour Size, mean ± SD (cm)	1.97 ± 0.83	1.70 ± 0.70 *	2.08 ± 0.86 *	1.85 ± 0.73 *	2.10 ± 0.90 *
Deceased, n (%)	58 (56%)	8 (27%) *	50 (68%) *	28 (48%) *	30 (67%) *
Recurrence, n (%)	31 (29%)	5 (17%) *	26 (36%) *	14 (24%) *	17 (38%) *

Abbreviations: BMI, Body Mass Index; SD, Standard Deviation. * Sample subgroup counts and metrics provided for illustration; actual distributions may vary.

**Table 2:** Best-performing Models for Tumour Grade AND Tumour Stage Prediction.

Target	Data	Classifier	AUC Mean	AUC 95% CI	Sensitivity	Specificity	F1 Score
Grade	TUMOUR + 10 mm PRF	MLPClassifier	0.961	[0.920, 1.000]	0.889	0.889	0.889
	Tumour	RandomForestClassifier	0.934	[0.891, 0.977]	0.867	0.867	0.863
	PRF 10 mm	CatBoostClassifier	0.900	[0.814, 0.986]	0.783	0.884	0.841
	PRF 15 mm	MLPClassifier	0.890	[0.825, 0.956]	0.806	0.806	0.802
	PRF 20 mm	LGBMClassifier	0.883	[0.825, 0.941]	0.764	0.819	0.798
	PRF 25 mm	RandomForestClassifier	0.876	[0.816, 0.937]	0.778	0.792	0.784
	PRF 30 mm	CatBoostClassifier	0.874	[0.813, 0.934]	0.806	0.847	0.827
STAGE	TUMOUR + 15 mm PRF	MLPClassifier	0.852	[0.790, 0.914]	0.776	0.776	0.772
	Tumour	MLPClassifier	0.831	[0.750, 0.911]	0.780	0.746	0.765
	PRF 15 mm	LogisticRegression	0.778	[0.704, 0.851]	0.702	0.667	0.682
	PRF 30 mm	ExtraTreesClassifier	0.771	[0.668, 0.874]	0.638	0.690	0.669
	PRF 25 mm	AdaBoostClassifier	0.759	[0.639, 0.879]	0.672	0.707	0.680
	PRF 10 mm	MLPClassifier	0.756	[0.657, 0.854]	0.679	0.643	0.654
	PRF 20 mm	MLPClassifier	0.711	[0.641, 0.781]	0.724	0.500	0.642

**Table 3:** DeLong tests for the best models in top-performing datasets.

	Model 1	Model 2	AUC 1	AUC 2	Z-Score	*p*-Value
Grade	Tumour + 10 mm PRF MLPClassifier	Tumour Random Forest Classifier	0.961	0.934	1.212807	0.225204
	Tumour + 10 mm PRF MLPClassifier	PRF 10 mm Cat Boost Classifier	0.961	0.9	2.416159	0.015685
	Tumour Random Forest Classifier	PRF 10 mm Cat Boost Classifier	0.934	0.9	1.234756	0.216921
Stage	Tumour + 15 mm PRF MLPClassifier	Tumour MLPClassifier	0.852	0.831	0.575251	0.565122
	Tumour + 15 mm PRF MLPClassifier	PRF 15 mm Logistic Regression	0.852	0.778	1.914466	0.055561
	Tumour MLPClassifier	PRF 15 mm Logistic Regression	0.831	0.778	1.339403	0.18044

## Data Availability

All relevant data are included within the manuscript. For further details, the corresponding author (A.A.M.) may be contacted upon reasonable request.

## Supplementary Materials

The following supporting information can be downloaded at: https://www.mdpi.com/article/10.3390/cancers17071220/s1. File S1. The CLEAR checklist.
